# Synthesis, characterization, and photocatalytic activities of green sol-gel ZnO nanoparticles using *Abelmoschus esculentus* and *Salvia officinalis*: A comparative study versus co-precipitation-synthesized nanoparticles

**DOI:** 10.1016/j.heliyon.2024.e24212

**Published:** 2024-01-09

**Authors:** Zakie Aalami, Mohammadsaleh Hoseinzadeh, Parsa Hosseini Manesh, Amir Hossein Aalami, Zarrin Es'haghi, Majid Darroudi, Amirhossein Sahebkar, Hasan Ali Hosseini

**Affiliations:** a Chemistry Department, Payame Noor University, 19395-4697, Tehran, Iran; b Department of Chemical Engineering, Faculty of Engineering, Ferdowsi University of Mashhad, Mashhad, Iran; c Department of Biology, Mashhad Branch, Islamic Azad University, Mashhad, Iran; d Department of Basic Medical Sciences, Neyshabur University of Medical Sciences, Neyshabur, Iran; e Biotechnology Research Center, Pharmaceutical Technology Institute, Mashhad University of Medical Sciences, Mashhad, Iran; f Applied Biomedical Research Center, Mashhad University of Medical Sciences, Mashhad, Iran

**Keywords:** ZnO nanoparticles, Removal methylene blue, Photocatalytic properties, Water treatment, *Salvia officinalis*, *Abelmoschus esculentus*

## Abstract

**Background:**

The development of green chemistry methods involving plant-based nanoparticle synthesis presents an affordable and eco-friendly approach for wastewater treatment and color removal. This study aimed to synthesize ZnO nanoparticles using the sol-gel method with *Salvia officinalis* and *Abelmoschus esculentus* plants, examining their photocatalytic efficiency for organic dye removal.

**Methods:**

To compare the properties of ZnO nanoparticles, another type of ZnO-NPs was synthesized using the co-precipitation method. The characterization of synthesized nanoparticles was performed using thermogravimetric analysis (TGA-DTG), X-ray diffraction (XRD), Dynamic Light Scattering (DLS), Zeta potential (ZP), field emission scanning electron microscopy (FE-SEM), Energy Dispersive X-ray (EDX), Fourier transform infrared spectroscopy (FTIR), and UV–Vis spectrophotometry.

**Results:**

Based on XRD results, the average crystalline size of nanoparticles was calculated using the Debye-Scherer equation for synthesized nanoparticles using the *S. officinalis* at 22.99 nm and for the *A. esculentus* at 29.79 nm, and for the co-precipitation method at 18.83 nm. The FE-SEM images showed spherical ZnO nanoparticles. Photocatalytic properties of ZnO-NPs were investigated for remove of methylene blue organic dye in the presence of UV light. The pH 10 was identified as the best pH, which had the highest percentage of color degradation. All three types of nanoparticles were tested by up to 360 min to optimize the dyeing time. For *A. esculentus*, the highest percentage of color removal occurred in the first 90 min (41.0 %), for *S. officinalis* nanoparticles between 75 and 90 min (86.9 %), and for chemically synthesized nanoparticles between 30 and 45 min (100 %).

**Conclusions:**

In conclusion, the best MB dye degradation capacity belonged to co-precipitation ZnO nanoparticles followed by *S. officinalis* and *A. esculentus* nanoparticles.

## Highlights

• ZnO-NPs synthesized via eco-friendly *S. officinalis*, *A. esculentus* & co-precipitation method.
• The UV–Vis, DLS, Zeta potential, FT-IR, XRD, TGA/DTG, FESEM, and EDX analyses confirmed the formation of stable ZnO-NPs.
• Photocatalysis applications of three types of ZnO-NPs have been reported via UV spectra.
• MB dye removal capacity decreased as follows: co-precipitated ZnO > *S. officinalis* > *A. esculentus.*

## Graphical abstract

**Figure:**
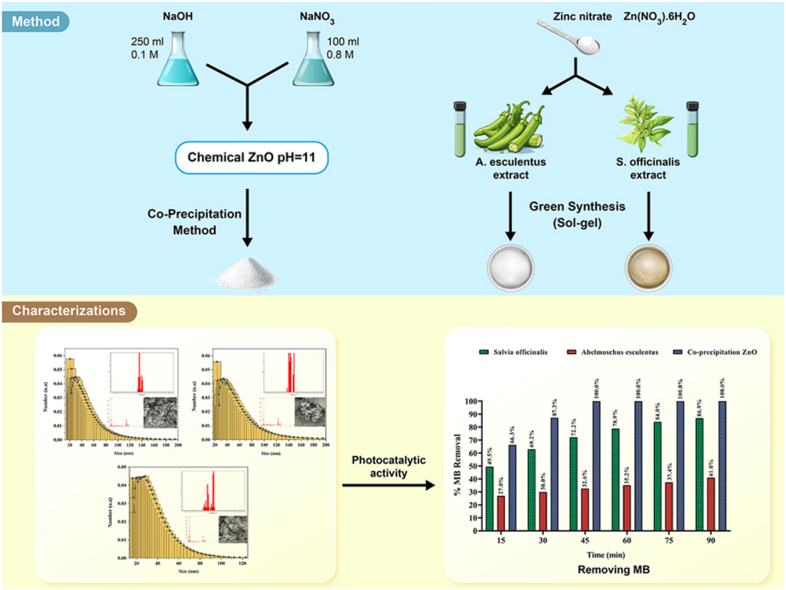

## 1 Introduction

Zinc oxide (ZnO) has emerged as a leading and effective candidate in the field of photocatalysis, demonstrating considerable potential in environmentally friendly approaches for green management systems [1]. Its distinctive position with a more negative conduction band and positive valence band among metal oxide photocatalysts makes ZnO the primary selection for restricting water and CO_2_ [2]. Various strategies, including green synthesis, doping or co-doping, creating nanocomposites, and forming heterojunctions with other semiconductors, offer viable pathways to modify the ZnO band structure. These alterations aim to enhance its redox capacity and ability to capture light, ultimately elevating its performance in photocatalysis [2]. Extensive research in this field has amplified the utilization of metal oxide nanoparticles as an advanced oxidation method in photocatalysis applications, garnering significant attention [3]. Zinc oxide nanoparticles (ZnO-NPs), as one of the essential metal oxide nanoparticles, are regularly applied in different fields due to their peculiar physicochemical and pharmaceutical features [4,5]. ZnO-NPs are low-cost, non-toxic, safe, biocompatible, mineralize more rapidly, exhibit higher efficiency, effectively produce H_2_O_2_, and possess a higher band gap of 3.37 eV, all contributing to their unique photocatalytic capabilities [6,7]. ZnO nanoparticles have been employed in several studies as antioxidants and also as antibacterial agents capable of restricting bacterial growth and acting as photocatalysts [[8], [9], [10]]. In recent advancements addressing water pollution and wastewater treatment, significant strides have been made in nanotechnology, particularly in the application of high-quality nanomaterials. Nanoparticles (NPs), characterized by their dimensions below 100 nm, exhibit remarkable chemical and physical properties that hold promise for water treatment processes.

Managing wastewater to ensure a safe water supply faces significant challenges, chiefly driven by the soaring demand for clean water [11]. Therefore, it is crucial to prioritize water treatment methods that primarily address remediation of water pollution issues. Efficient wastewater treatment systems, capable of yielding safe water with minimal preparation time, are urgently needed. Various traditional treatment approaches have been employed, including biological treatment, membrane separation, chemical precipitation, electrochemical techniques, adsorption, and others [12,13]. However, these methods come with inherent challenges and limitations during the cleanup process. Issues such as high operational costs, inefficiency at low pollutant concentrations, lack of selectivity, and the potential release of harmful substances into the aquatic environment have been observed [14,15].

The mechanism of dye degradation entails the transfer of electrons from the nanoparticle to oxygen species, biomolecules, or adjacent organic compounds. For this reason, ZnO-NPs prove to be a practical and efficient choice as a photocatalyst for the degradation of dyes present in wastewater.

Many papers demonstrated the role of ZnO-NPs in the dye removing and also water treatment [[16], [17], [18], [19], [20]]. Our study, originating from a Master's thesis with registration number 1221/8359, extensively investigates the photocatalytic capabilities of ZnO-NPs. These nanoparticles were synthesized using green sol-gel techniques, utilizing aqueous extracts from *Salvia officinalis (S. officinalis)* and *Abelmoschus esculentus (A. esculentus)*. We compared these with ZnO-NPs synthesized via the co-precipitation method for potential application in dye removal, such as Methylene blue. Subsequently, the synthesized ZnO-NPs underwent comprehensive characterization employing UV–VIS spectroscopy, DLS, Zeta potential, TGA/DTA, XRD, FTIR, FESEM, and EDX analyses.

## 2 Methods and materials

### 2.1 Preparation of green ZnO-NPs applying the sol-gel method

Fresh *A. esculentus* and *S. officinalis* were collected from Khorasan Province, Iran. 100 g of each plant was added to 100 mL of double-distilled water (DDW) separately. Then, they were placed on the heater stirrer at 60 °C for 4 h. The *A. esculentus* extraction was filtered by Whatman #1. Since the *S. officinalis* solution was not homogeneous, it was centrifuged at 5000 rpm for 5 min to obtain a completely homogeneous and clear solution, and then the extract was filtered by Whatman #1.

Next, 40 ml of both pale-yellow extracts were placed in the water bath at 50 °C and 250 rpm. Subsequently, 7 g of Zn (NO_3_)_2_ · 6H_2_O (Sigma Aldrich, CAS Number 10196-18-6) is dissolved in 20 mL of DDW, which is then added to the extract in 5 steps (every 5 min). In this step, no precipitate was formed, and the solution looked dense. Finally, the volume of the solution was increased to 100 mL, called the Sol solution. Afterward, the temperature increased to 80 °C and was stirred for 6 h. Next, 10 g of the solution was put in the oven for 4 h at 90 °C to evaporate the water. After water evaporation, the gel was obtained. Following the synthesis of zinc oxide, the solution was calcined for 2 h at 600 °C. The increasing rate of temperature was set at 3 °C per minute. After 2 h, pale-yellow powders were obtained.

#### 2.1.1 Sol-gel synthesis mechanism

##### 2.1.1.1 Hydrolysis

M-OR + HOH → M-OH + R-OH

##### 2.1.1.2 Condensation

（1）M-OH + OH-M → M-O-M + H_2_O (2) M-OR + M-OH → M-O-M + M-OH

### 2.2 Preparation of ZnO-NPs applying the co-precipitation method

The co-precipitation process for ZnO-NPs involves utilizing Zn (NO_3_)_2_. 6H_2_O and NaOH as primary materials. Initially, a 250 ml 0.1 M sodium nitrate solution and a 100 ml 0.8 M NaOH solution are prepared separately. These solutions are individually poured into a beaker containing a magnet, placed on a magnetic stirrer, and allowed to stir at 300 rpm for about an hour. Subsequently, the pH of the zinc nitrate solution is measured at 5, followed by the gradual addition of 0.8 M NaOH dropwise to the stirred Zn (NO_3_)_2_ solution for 45–60 min pH 11 is identified as the optimal condition for sedimentation. The reaction is allowed to continue for 2 h after NaOH addition to complete the co-precipitation reaction. The solution container is covered and kept in a dark place for 48 h to prevent any light-induced chemical alterations. Exposure to light during this phase can potentially trigger photochemical reactions that might interfere with the synthesis process or impact the solution's characteristics.

After 48 h, the solution is slowly poured over the sediment, and the remaining solution and sediment are centrifuged for 10 min. The upper solution is decanted, and the sediment is washed with distilled water before undergoing another 10-min centrifugation. This washing and centrifugation process is repeated three times. Following the third cycle, the sediment is washed with ethanol, and another round of centrifugation is performed to eliminate waste particles adhering to the nanoparticles. The sediment, separated from the bottom of the tube using ethanol, is transferred to a container and placed in an oven at 60 °C for 3 h. The heat treatment in the oven ensures the complete conversion of Zn (OH)_2_ to ZnO during the drying process.

#### 2.2.1 Co-precipitation synthesis mechanism

##### 2.2.1.1 Precipitation

• Zinc nitrate solution is mixed with a base, typically sodium hydroxide (NaOH) or ammonium hydroxide (NH_4_OH).
• The addition of the base results in the precipitation of zinc hydroxide:

Zn(NO_3_)_2_ + 2NaOH → Zn(OH)_2_ + 2NaNO_3_

##### 2.2.1.2 Conversion to ZnO

• Similar to the sol-gel method, heating the zinc hydroxide precipitate leads to the conversion into zinc oxide nanoparticles:

Zn(OH)_2_ → ZnO + H_2_O

### 2.3 Characterization of ZnO-NPs

The ZnO-NPs were examined using the following methods: DLS (Cordouan, Vasco3, France), UV-2550 Spectrophotometer (Shimadzu 2600i, Japan), TGA-DTG (TGA-50 Series, Shimadzu, Japan), FESEM and EDX (TESCAN, MIRA 3, Brno, Czech Republic), X-Ray diffraction (D8 ADVANCE, Bruker, USA), Fourier-transform infrared spectroscopy (Thermo Nicolet AVATAR (Shimadzu, Japan)), and Zeta potential (Zetasizer Nano ZS (Malvern Instruments, UK).

### 2.4 Methylene blue (MB) preparation

The chemical formula of methylene blue (MB) dye is C_16_H_18_ClN_3_S, and the molecular weight is 319.85 g/mol [21]. It is a dark green, odorless, solid powder at room temperature [22]. When dissolved in water, a blue solution is obtained. Its aqueous form has three water molecules per unit of methylene blue. To prepare a solution with a 10^−3^ M concentration of MB, we first weighed the powder with a digital scale according to the calculations of 0.0159 g and the volume of it in the 50 cc balloon. Then cover the balloon thoroughly with aluminum foil and keep it in the refrigerator, and each time, work with MB from this solution in 1 cc with a pipette and pour it into 100 cc balloons with a volume (10^−5^ M). By diluting, the weighing error reaches its minimum value. Then calibrate the UV spectrophotometer, adjust the wavelength between 400 and 800 nm, and take a 10^−5^ M MB solution.

### 2.5 Photocatalytic application of synthesized ZnO-NPs

The parameters of removing dyeing time, number of nanoparticles, and pH were investigated and optimized. First, to optimize the color removal time, a specific amount of these ZnO-NPs in a certain amount of MB pigment (1:100 ratio) was placed under the UV lamp for 3 h, and it was found that the maximum color removal was done in the first 75–90 min. Therefore, the rest of the ratios were examined in 90 min. Solutions with different pigment ratios to the ZnO-NPs (1:50, 1:100, and 1:150) were prepared to optimize the nanoparticle concentration. The ultrasonic device dispersed all these solutions in about 60 min. Then these solutions were placed one by one on the stirrer at a speed of 250 rpm under the ultraviolet lamp radiation, and then their absorption spectrum was taken by the UV spectrophotometer in the wavelength range of 400–800 nm. The color removal percentage is calculated for each of the following equations:

(1) $$\%\;\text{MB}\;\text{colur}\;\text{removal}=\frac{A0\;-\;\text{At}}{A0}\times 100$$

where A_0_ and A_t_ (both in mgL^−1^) are the initial and remaining concentration of MB in solution, respectively, and A_t_ is the absorbance at any irradiation time t (min).

Moreover, to optimize the effect of pH, prepared a 10^−5^ M solution of MB pigment and nanoparticles with a ratio of 1:100 and using 1 M HCl and 1 M NaOH solutions; the pH of the three solutions containing the pigment and nanoparticles was set to 4, 7, and 10. Then, each solution was dispersed for 1 h and stirred at 250 rpm under the light of an ultraviolet lamp—the absorption spectrum by an ultraviolet–visible spectrophotometer. Acids and bases are added drop by drop to maintain the concentration of the primary pigment and nanoparticle solution.

After optimizing the concentration, pH (at 10), and irradiation time, we prepare a solution with a 1:100 ratio of pigment to salvia zinc oxide nanoparticles. Then, a 10^−5^ M MB solution was prepared, and its absorption spectrum was measured with an ultraviolet–visible spectrophotometer. After that, 0.00814 g of ZnO-NPs were added to the beaker containing 10^−5^ M MB and dispersed for 1 h. Afterward, we obtained a pH of 10 for the solution with NaOH. Subsequently, the dispersed solution was stirred at 250 rpm under the UV lamp for about 75–90 min to become decolorized. The absorption spectrum of this solution is taken every 15 min to see the gradual fading of the solution.

### 2.6 Statistical analysis

The data analysis was conducted using Statistical Package for the Social Sciences (SPSS) version 27 by IBM Inc. (Chicago, IL, USA). All tests were performed in triplicate, and the results are presented as mean values along with standard deviation (SD). Graphs were generated using GraphPad Prism 9.4 by GraphPad Software Inc. (USA) and Origin Pro 2022 by Origin (Germany). Python 3.11.0 was utilized for band-gap energy calculations. A significance level of p < 0.05 was chosen to determine statistical significance.

## 3 Results

### 3.1 UV–Vis spectrophotometer

**Fig. 1:**
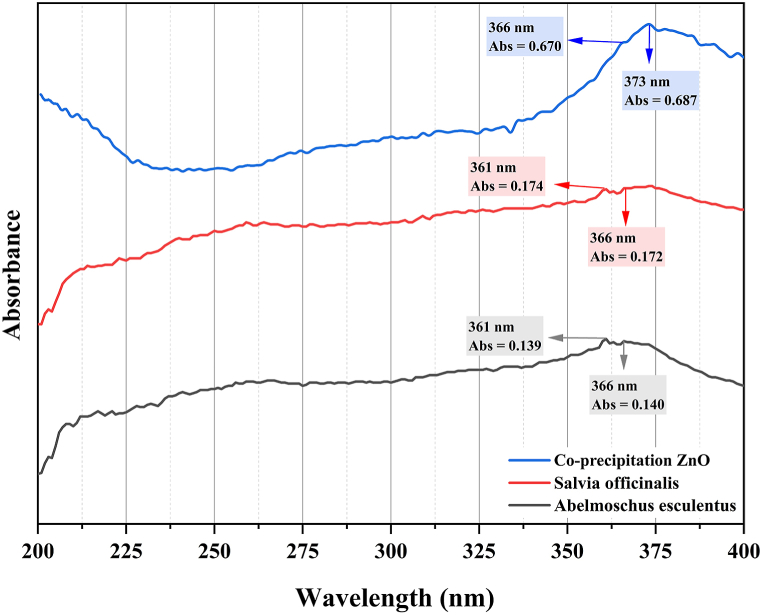
The UV–Vis absorption spectrum confirming the presence of ZnO-NPs.

The produced ZnO-NPs were primarily characterized using the Shimadzu 1280 UVVis spectrophotometer. The double-distilled water was used to disperse the ZnO-NPs. All peaks between 340 and 380 nm verify the production of ZnO NPs, for which we measured the peak for *S. officinalis* at 361 nm (Abs. 0.174), for *A. esculentus* at 366 nm (Abs. 0.140), and for co-precipitation ZnO-NPs obtained at 373 nm (Abs. 0.687), respectively (Fig. 1). To determine the band-gap energy utilizing the Tauc method, the Python software 3.11.0 was utilized. This involved converting the wavelength from nanometers to electron volts (eV) and generating a plot of (αhν)^2^ against hν.

The Tauc equation is:

(2) $$\alpha hv={A\;\left(hv-E_{g}\right)}^{n}$$

Convert wavelength to photon energy (eV) formula is:

(3) $$E=\frac{A=1240}{Wavelenght}$$

Calculate the square of the product (αhν)^2^ is:

(4) $${\alpha hv}^{2}={\left(Absorbance\times E\right)}^{2}$$

where.

• α is the absorption coefficient.
• hν is the photon energy (eV).
• A is a constant = 1240
• Eg is the band-gap energy.
• n depends on the nature of the electronic transition (often taken as 1/2 or 2 for direct and indirect transitions, respectively).

**Fig. 2:**
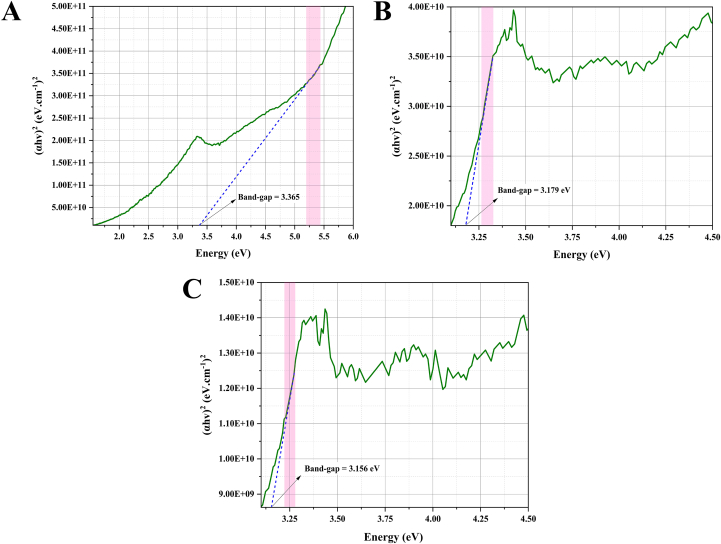
Based on the Tauc equation, the band-gap energy was calculated as follows: for for co-precipitation ZnO, 4.287 eV (A); *S. officinalis* ZnO-NPs, 3.179 eV (B); and for *A. esculentus* ZnO-NPs, 3.156 eV (C).

The plot of (αhν)^2^ against hν will yield a straight line, and the band-gap energy (Eg) can be estimated from the intercept on the energy axis. Based on the Tauc equation, the band-gap energy was calculated as follows: for for co-precipitation ZnO, 3.365 eV (Fig. 2A); *S. officinalis* ZnO-NPs, 3.179 eV (Fig. 2B); and for *A. esculentus* ZnO-NPs, 3.156 eV (Fig. 2C).

#### 3.1.1 X-ray diffraction (XRD)

The X-ray diffraction (XRD) was carried out by utilizing D8 ADVANCE, Bruker Company (USA), working at optimized conditions. Debye Scherrer's equation was used to calculate the average size of synthesized ZnO-NPs. The diffraction peaks of XRD were assessed in the range of 2θ = 10–80°.

Scherrer's formula is:

(5) $$D=\frac{K\times \lambda }{\beta \times \mathrm{Cos}\;\theta }$$

where:

D is the average crystallite size.

K is the Scherrer's constant (0.89).

λ is the X-ray wavelength (0.15406 nm)

β is the full width at half maximum (FWHM) of the diffraction peak in radians.

θ is the Bragg angle.

**Fig. 3:**
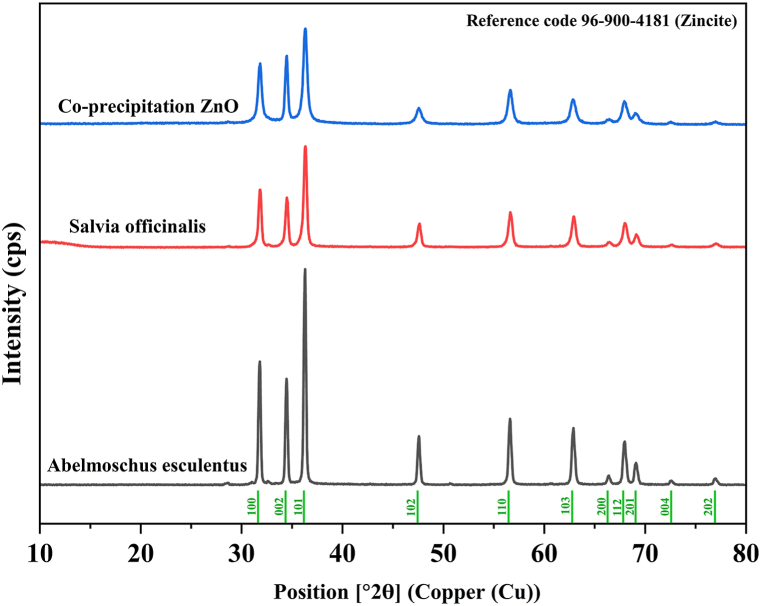
The XRD pattern indicating the presence of ZnO peaks.

The crystalline sizes for (1 0 0), (0 0 2), (1 0 1), (1 0 2), (1 1 0), (1 0 3), (1 1 2), and (2 0 1), were found to have peaks of 25.47 nm, 26.84 nm, 24.90 nm, 22.98 nm, 20.96 nm, 20.94 nm, 21.04 nm, and 20.84 nm for *S. officinalis*, respectively, and the average was calculated as 22.99 nm (Table 1, Fig. 3).

**Table 1:** Obtained results XRD pattern of ZnO-NPs.

S. officinalis	***hkl***	**dhkl (Å)**	**Peak position 2θ (°)**	**FWHM**	**Crystallite Size (nm)**
	1 0 0	2.809	31.827	0.339	25.47
	0 0 2	2.598	34.491	0.324	26.84
	1 0 1	2.471	36.316	0.351	24.90
	1 0 2	1.908	47.608	0.395	22.98
	1 1 0	1.623	56.658	0.45	20.96
	1 0 3	1.475	62.927	0.465	20.94
	1 1 2	1.377	68.012	0.476	21.04
	2 0 1	1.357	69.147	0.484	20.84
A. esculentus	***hkl***	**dhkl (Å)**	**Peak position 2θ (°)**	**FWHM**	**Crystallite Size (nm)**
	1 0 0	2.812	31.208	0.27	31.98
	0 0 2	2.600	33.928	0.267	32.56
	1 0 1	2.474	35.648	0.27	32.36
	1 0 2	1.910	46.968	0.294	30.86
	1 1 0	1.624	55.968	0.324	29.11
	1 0 3	1.477	62.248	0.357	27.26
	1 1 2	1.378	67.368	0.378	26.49
	2 0 1	1.358	68.688	0.364	27.69
Co-precipitation ZnO	***hkl***	**dhkl (Å)**	**Peak position 2θ (°)**	**FWHM**	**Crystallite Size (nm)**
	1 0 0	2.810	31.814	0.422	20.46
	0 0 2	2.601	34.451	0.317	27.42
	1 0 1	2.472	36.303	0.456	19.16
	1 0 2	1.910	47.564	0.577	15.73
	1 1 0	1.623	56.631	0.522	18.07
	1 0 3	1.477	62.855	0.589	16.52
	1 1 2	1.378	67.954	0.565	17.72
	2 0 1	1.358	69.076	0.648	15.56

For *A. esculentus*, the crystalline sizes for (1 0 0), (0 0 2), (1 0 1), (1 0 2), (1 1 0), (1 0 3), (1 1 2), and (2 0 1), peaks were found to be 31.98 nm, 32.56 nm, 32.36 nm, 30.86 nm, 29.11 nm, 27.26 nm, 26.49 nm, and 27.69 nm, respectively, and the average was calculated as 29.79 nm (Table 1, Fig. 3).

For co-precipitate ZnO with the same peaks, the crystalline sizes were calculated as 20.46 nm, 27.42 nm, 19.16 nm, 15.73 nm, 18.07 nm, 16.52 nm, 17.72 nm, and 15.56 nm, respectively, and the average was calculated as 18.83 nm (Table 1, Fig. 3).

#### 3.1.2 Fourier transform infrared spectroscopy (FTIR)

**Fig. 4:**
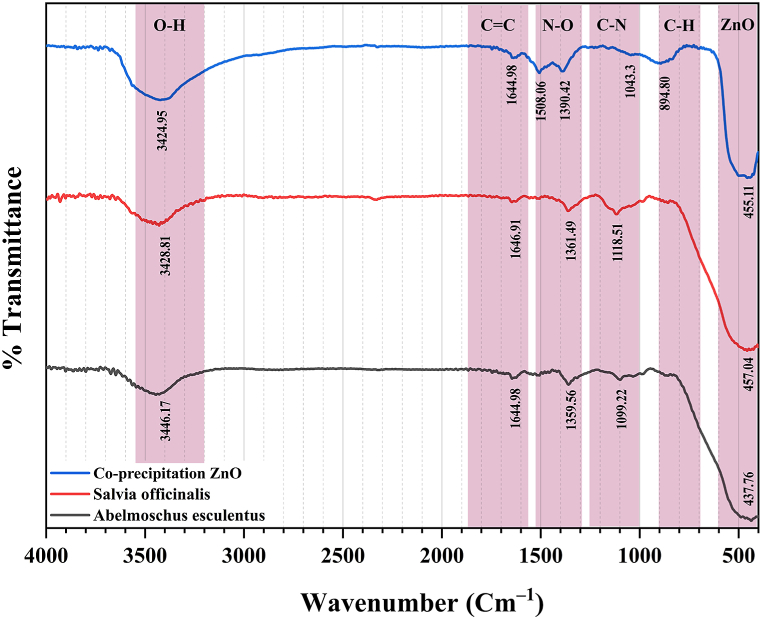
The FTIR charts of ZnO-NPs.

The surface chemistry of ZnO-NPs was characterized by Fourier transform infrared spectroscopy (FTIR) (Thermo Nicolet Avatar 370, USA). The functional groups attached to the surface of NPs were detected in the range of 4000–400 cm^−1^ (Fig. 4). The peak at 400–600 cm^−1^ corresponds to the ZnO. Numerous FTIR peaks observed on all spectra in the 1700–600 cm^−1^ range can be assigned to C=O, C–O, C–C, and C–H vibrations, respectively. A broad maximum observed in the 3200–3550 cm^−1^ range is attributed to the stretching vibration of hydroxyl compounds (adsorbed water).

#### 3.1.3 Dynamic Light Scattering (DLS)

Dynamic Light Scattering (DLS) analysis enables us to accurately determine the size distribution profiles of sub-micron particles. This method is very effective for investigating the function of nanoparticles in suspensions. DLS analyses for these three NPs were performed with the conditions of 50 % laser power, a wavelength of 657 nm, a 1.36 M refractive index, and a 1.197 viscosity at 25 °C.

**Fig. 5:**
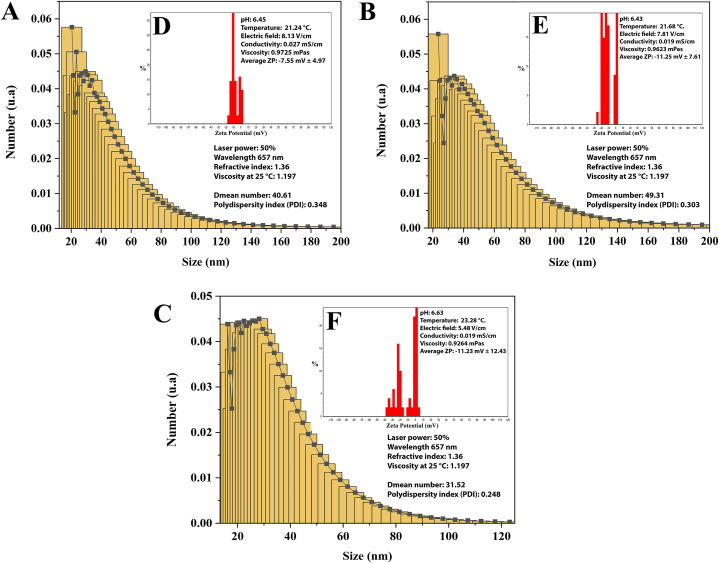
Zeta potential and size distribution of ZnO-NPs as analyzed by DLS. (A) *S. officinalis* DLS pattern; (B) *A. esculentus* DLS pattern; and (C) co-precipitation ZnO DLS pattern. The zeta potential graph of ZnO-NPs (D–F). (D) zeta potential of *S. officinalis*; (E) zeta potential of *A. esculentus*; and (F) zeta potential of co-precipitation ZnO.

For *S. officinalis*, the measurements of mean intensity, mean volume, and mean number were determined at 204.11, 198.55, and 40.61, respectively, with the polydispersity index (PDI) at 0.348, which indicates the size distribution (Fig. 5A).

For *A. esculentus*, the DLS, mean intensity, mean volume, and mean number were obtained at 208.85, 216.76, and 49.31, respectively, with the PDI at 0.303 (Fig. 5B).

For co-precipitate ZnO-NPs, the DLS was calculated at mean intensity, mean volume, and mean number at 114.03, 74.18, and 31.52, respectively, with the PDI at 0.248 (Fig. 5C).

#### 3.1.4 Zeta potential (ZP)

Nanoparticles with zeta potentials between −10 and + 10 mV are neutral, but those with zeta potentials higher than +30 mV or lower than −30 mV are highly cationic and strongly anionic, respectively. ZnO-NPs were diluted in free deionized water containing a final sodium chloride concentration of 1 mM.

The zeta potential was determined using a Zetasizer Nano ZS (Malvern Instruments, UK) with the following parameters for *S. officinalis*: 8.13 V/cm electric field, 0.027 mS/cm conductivity, and 0.9725 mPas viscosity at 21.24 °C. The average zeta potential of *S. officinalis* ZnO-NPs at pH 6.45 was −7.55 mV with a standard deviation of 4.97 (Fig. 5D). The ZP was determined for *A. esculentus* with the following conditions: 7.80 V/cm electric field, 0.019 mS/cm conductivity, and 0.9623 mPas viscosity at 21.68 °C. The average zeta potential of *A. esculentus* ZnO-NPs at pH 6.43 was −15.27 mV with a standard deviation of 9.02 (Fig. 5E).

The ZP was determined for co-precipitate ZnO-NPs with the following criteria: 5.48 V/cm electric field, 0.019 mS/cm conductivity, and 0.9264 mPas viscosity at 23.28 °C. The average zeta potential of ZnO-NPs co-precipitation at pH 6.63 was −11.23 mV with a standard deviation of 12.43 (Fig. 5F).

#### 3.1.5 TGA and DTG analysis

**Fig. 6:**
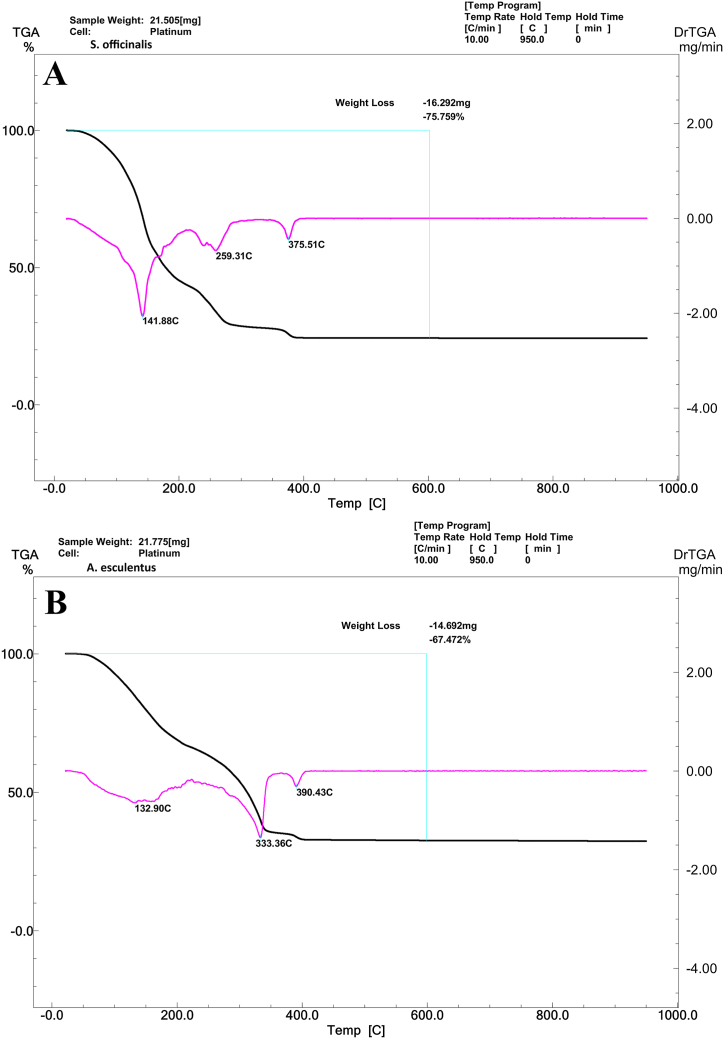
The TGA/DTG diagram of *S. officinalis* ZnO-NPs (A) and *A. esculentus* ZnO-NPs (B).

Thermogravimetric analysis (TGA) measured losing or gaining weight as a temperature function. Derivative Thermogravimetry (DTG) is a technique for recording the difference in temperature between a substance and a reference material as a function of time or temperature. The TGA was performed on salvia gel from 25 to 950 °C (Fig. 6A). At the start of the analysis, the mass of the synthesized sample decreases gradually with increasing temperature. Water molecules evaporate at a temperature of 141.88 °C. The following breakdown of the DTG chart is at 259.31 °C, which is related to the burning of organic molecules and their weight loss. At the temperature of 375.51 °C, another break can be seen in the graph: the crystallization temperature, which shows that zinc oxide crystals are formed from this temperature onwards. It indicates that the samples synthesized at high temperatures (up to 1000 °C) are also thermally stable (Fig. 6A).

TGA was conducted on okra gel within a temperature range of 25–950 °C, and evaporation involves converting water molecules into a gaseous state, which occurs at a temperature of 132.90 °C. The present analysis pertains to the DTG chart, wherein the temperature of 333.36 °C indicates the combustion of organic molecules and the consequent reduction in their mass (Fig. 6B). At a temperature range of 390–400 °C, the graph displays an additional inflection point indicating the onset of crystallization temperature. This phenomenon signifies the formation of zinc oxide crystals beyond this temperature threshold. The findings suggest that the specimens produced at elevated temperatures (reaching 1000 °C) exhibit exceptional thermal stability.

#### 3.1.6 FE-SEM and EDX

**Fig. 7:**
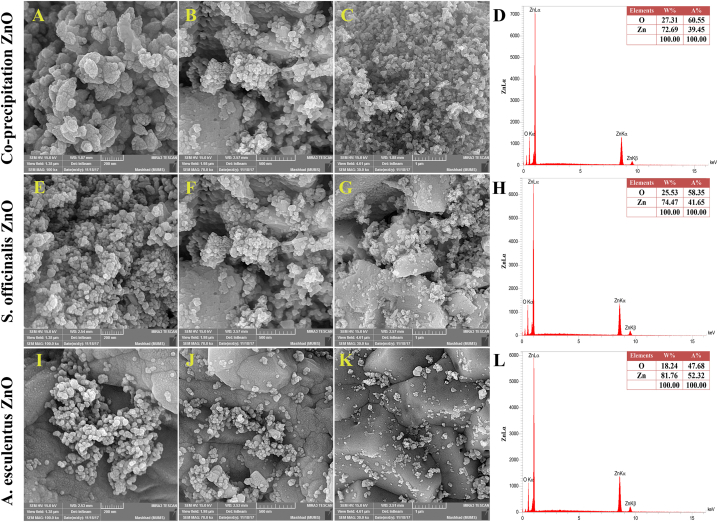
The FESEM and EDX of ZnO-NPs. (A–C) co-precipitation ZnO images (200 nm, 500 nm, and 1000 nm) respectively; (D) EDX pattern of co-precipitation ZnO; (E–G) *S. officinalis* images (200 nm, 500 nm, and 1000 nm) respectively; (H) EDX pattern of *S. officinalis* ZnO; (I–K) *A. esculentus* images (200 nm, 500 nm, and 1000 nm) respectively; (L) EDX pattern of *A. esculentus* ZnO.

Field Emission Scanning Electron Microscopy (FE-SEM), coupled with energy-dispersive X-ray spectroscopy (EDX) (MIRA3 TESCAN operating at 15 kV), was employed to capture micrographs of the synthesized ZnO-NPs. As per the scale located at the bottom of the image, the average sizes of the ZnO-NPs synthesized using the co-precipitation method (Fig. 7 A–C), *S. officinalis* (Fig. 7 E–G), and *A. esculentus* (Fig. 7 I–K) were approximately 50 nm, 30 nm, and 40 nm, respectively. In the case of co-precipitated ZnO, EDX analysis revealed oxygen accounting for 27.31 % of the total weight and zinc for 72.69 % (Fig. 7 D). For *S. officinalis*, the weight percentage of oxygen was 25.53 %, and that of zinc was 74.47 % (Fig. 7H). In the case of *A. esculentus*, the weight percentage of oxygen stood at 18.24 %, while that of zinc was 81.76 %, indicating the absence of impurities within these nanoparticles (Fig. 7 L).

### 3.2 Investigating the photocatalytic properties of synthesized ZnO-NPs

**Fig. 8:**
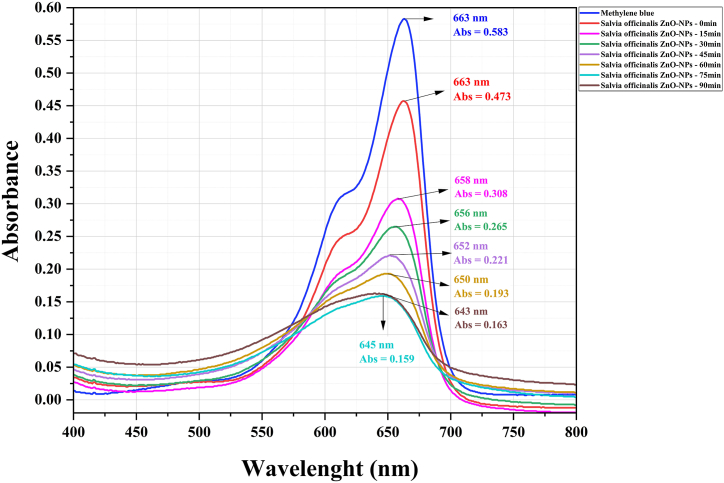
The UV-VIS absorption spectrum of the 1:100 ratio of MB pigment to *S. officinalis* ZnO-NPs through the green sol-gel method at pH 10.

**Fig. 9:**
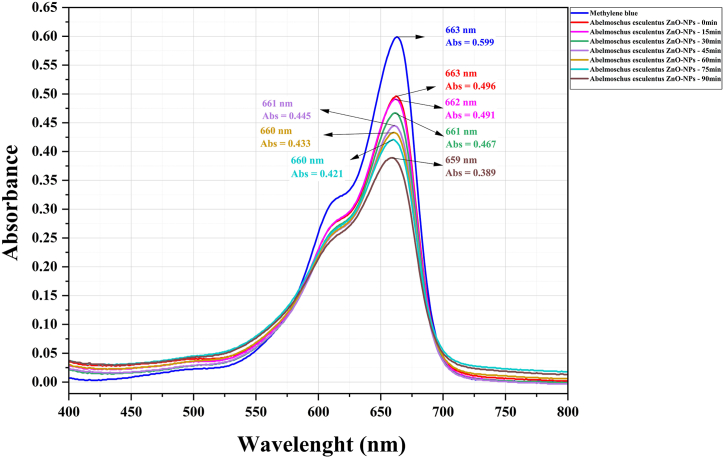
The UV-VIS absorption spectrum of the 1:150 ratio of MB pigment to *A. esculentus* ZnO-NPs through the green sol-gel method at pH 10.

**Fig. 10:**
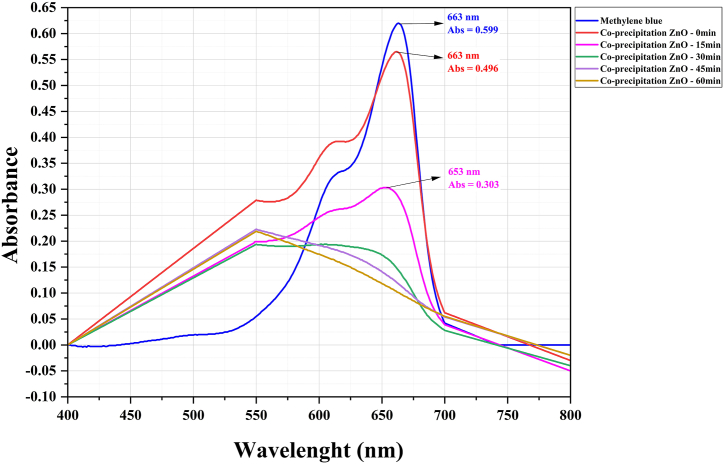
The UV-VIS absorption spectrum of the 1:100 ratio of MB pigment to ZnO synthesized by the chemical co-precipitation method at the pH 10.

Solutions with varied pigment ratios for the ZnO-NPs (1:50, 1:100, and 1:150) were prepared to optimize the nanoparticle concentration. Our findings revealed that the optimal ratio for *S. officinalis* and co-precipitation ZnO was 1:100, whereas for *A. esculentus*, the most effective ratio was 1:150. Notably, a pH level of 10 exhibited the highest efficacy in pigment degradation. This can be attributed to the abundance of OH hydroxyl radicals in alkaline solutions. Absorption spectra were conducted for the optimized ratio of 1:100 MB pigment to ZnO-NPs at pH = 10, post-calcination at 600 °C. Fig. 5, Fig. 6, Fig. 7 illustrate a gradual reduction in pigment intensity due to UV light radiation, leading to near-complete removal, resulting in a nearly colorless solution. Specifically, *S. officinalis* ZnO-NPs displayed decolorization percentages of 84.2 %, 89.3 %, and 87.41 % (Fig. 8). For *A. esculentus* ZnO-NPs, the experiment yielded pigment removal percentages of 41 %, 49 %, and 35 % across three repetitions (Fig. 9). Notably, co-precipitation ZnO exhibited complete color removal after 45 min (Fig. 10).

**Fig. 11:**
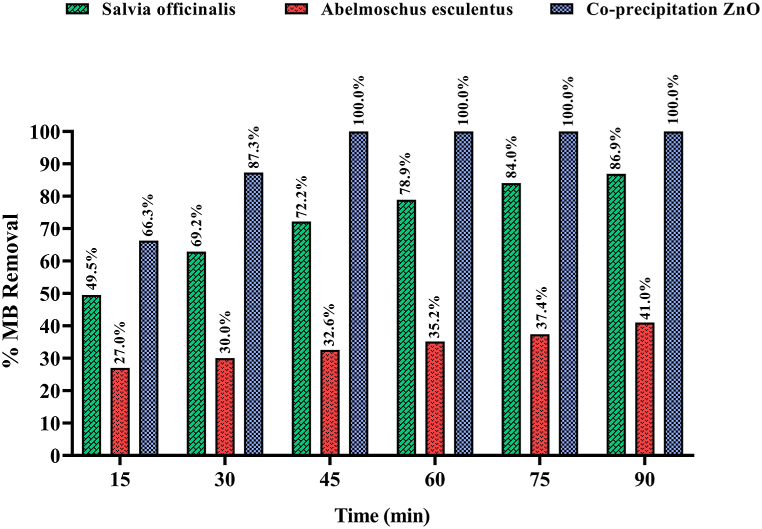
The effect of using zinc oxide nanoparticles on MB decolorization at different times. The results show that using *A. esculentus* ZnO-NPs, 41.0 % discoloration is observed in MB after 90 min. For *S. officinalis*, the most significant difference in MB removal is observed between 0 and 15 min, 15–30 min, and 30–45 min. Comparing the average decolorization effects of *S. officinalis* NPs shows more speed and power than *A. esculentus* ZnO-NPs. Co-precipitation ZnO-NPs showed the most significant effect of MB removal in the first 45 min (100 %), which is not significantly different from the later times.

Our findings demonstrated that the utilization of *S. officinalis* ZnO-NPs notably augmented the average removal of MB dye, with this effect becoming more pronounced over time. Upon comparing averages, the most effective color removal was observed at 90 min (86.9 %). However, according to statistical analysis (Duncan's test), there's no significant difference between the 75-min (84.0 %) and 90-min results, indicating a stable color removal process. Notably, the most noticeable differences in color removal were observed between the 0–15 min (49.5 %), 15–30 min (62.9 %), and 30–45 min (72.2 %) intervals, suggesting a relatively slower progression (Fig. 11). The outcomes illustrate that the use of chemical co-precipitation synthesized ZnO-NPs led to a rapid escalation in MB dye removal during the experiment, achieving complete removal after approximately 45 min (Fig. 11). Conversely, the least efficient dye removal was observed with *A. esculentus*. Our results indicate that okra NPs induced minimal discoloration of MB, with only around 41 % observed after 90 min (Fig. 11).

## 4 Discussion

In this research, ZnO nanoparticles were synthesized using *A. esculentus* and *S. officinalis* plants using the sol-gel technique and ZnO by chemical co-precipitation method. Then the photocatalytic properties of these nanoparticles were investigated by removing the MB organic pigment. As observed, in general, the decolorization effect was more and more robust in the chemical method, and after that, the nanoparticle method synthesized in *S. officinalis* had a higher and stronger decolorization effect than *A. esculentus*.

Chen et al. [23] used *Scutellaria baicalensis* (*S. baicalensis*) root extract to synthesize of ZnO-NPs. The characterization of the nanoparticles was determined through the use of UV–vis, FTIR, EDX, FE-TEM, and XRD. The findings of the characterization analysis indicate that the phytochemicals present in the extract of *S. baicalensis* exhibit the ability to function as a reducing agent during the formation of nanoparticles, which are observed to be approximately 50 nm in size and possess a spherical morphology. Moreover, the photocatalytic potential of Sb–ZnO nanoparticles was investigated as a photocatalyst. This study investigates the catalytic removal of methylene blue by utilizing Sb–ZnO nanoparticles under UV irradiation. The Sb–ZnO nanoparticles were synthesized through the absorbance of degraded dye solution. The results indicate that Sb–ZnO nanoparticles possess significant photocatalytic potential, as evidenced by the removal of 98.6 % of MB solution with a concentration of 0.05 mg/ml under UV irradiation over 210 min.

Selvaraj et al. [24] synthesized pure and Gd-doped ZnO using a facile co-precipitation method, as reported in their study. The samples that were made ready for examination underwent analysis via various techniques, including X-ray diffraction (XRD), ultraviolet–visible diffuse reflectance spectroscopy (UV-DRS), Fourier transforms infrared spectroscopy (FTIR), scanning electron microscopy (SEM), X-ray photoelectron spectroscopy (XPS), and transmission electron microscopy (TEM). The removal of MB dye was carried out via photocatalysis using visible light irradiation. The removal efficiency of Gd-doped ZnO is superior to that of pristine ZnO. The experimental results indicate that the incorporation of 3 % gadolinium into zinc oxide leads to a significant enhancement in the removal of MB dye, with a recorded efficiency of 93 % under visible light irradiation for a duration of 90 min.

Utilizing components of *Euphorbia milii* (*E. milii*) leaves, Venkatesan et al. prepared ZnO-NPs [25]. The SEM images revealed the existence of spherical ZnO-NPs, while TEM images demonstrated the uniformity of the ZnO-NPs with diameters ranging from 12 to 20 nm. According to the Brunauer-Emmett-Teller (BET) analysis, it was determined that the ZnO-NPs possess a specific surface area of 20.46 m^2^/g, along with a pore diameter ranging from 2 nm to 10 nm and a pore volume of 0.908 cm^3^/g. The EDAX analysis demonstrated the presence of Zn and O elements and the absence of impurities, thereby confirming the pristine nature of the ZnO-NPs. The XRD analysis revealed the presence of distinct crystalline peaks that corresponded to the hexagonal wurtzite structure of ZnO. The average size of the crystallites was determined to be 16.11 nm. The FTIR spectrum exhibited pronounced absorption peaks at 512 cm^−1^ and 534 cm^−1^, associated with ZnO. The ZnO-NPs demonstrated significant removal of MB dye through photocatalytic activity under natural sunlight exposure. The % removal efficiency of 98.17 % was attained at an illumination duration of 50 min.

In another study, Venkatesan et al. [26] conducted a study wherein they utilized *Solanum trilobatum* (*S. trilobatum*) leaf extract as a reducing agent to synthesize ZnO-NPs. The SEM images revealed the formation of spherical ZnO nanoparticles with an average diameter of approximately 25 nm. The FTIR spectrum of ZnO NPs exhibited a prominent peak at 540.94 cm^−1^, attributed to the distinctive stretching of the Zn–O bond. The concentration of MB dye experienced an exponential decrease due to the presence of ZnO NPs catalysts. Consequently, a photocatalytic property effectively removed 94.07 % of MB dye within 90 min of sunlight illumination. The present study posits that the observed photocatalytic activity can be attributed to the suitable band gap energy, high crystallinity, and diminutive particle size of ZnO nanoparticles.

In a different study, Soto-Robles et al. [27] employed a green route method to synthesize ZnO nanoparticles in varying concentrations (1, 2, 4, and 8 % w/v) using an extract of *Justicia spicigera*. The specimens underwent characterization by applying FT-IR and UV–vis spectrophotometric techniques. The characterization process was also supplemented by utilizing XRD, SEM, and TEM. Ultimately, the various catalysts were examined regarding their efficacy in facilitating the removal of MB when exposed to ultraviolet light. The samples exhibited the characteristic Zn–O bond band at a wavenumber of 618 cm^−1^. The formation of ZnO-NPs was confirmed through UV–vis studies, which exhibited a distinctive signal at 373–376 nm. This research investigates the photocatalytic activity of ZnO-NPs that were biosynthesized using various concentrations (1 %, 2 %, 4 %, and 8 %) of *Justicia spicigera* extract. The removal of MB dye in an aqueous medium under UV radiation is used for analysis. The experimental results indicate that after 30 min of stirring in the absence of light, the samples M1-ZnO, M2-ZnO, M3-ZnO, and M4-ZnO exhibited MB adsorption percentages of approximately 11 %, 15 %, 16 %, and 18 %, respectively. The observed outcomes can be attributed to the extract on the exterior of the biosynthesized ZnO-NPs. This can be explained by the electrostatic attraction between the extract, which carries negative charges, and the MB molecules, which carry positive charges. After a duration of 90 min of irradiation, the photocatalysts M1-ZnO, M2-ZnO, M4-ZnO, and M8-ZnO exhibited photocatalytic removal results of 73.44 %, 81.92 %, 83.05 %, and 92.78 %, respectively, concerning the removal of MB. The findings suggest that the M8-ZnO specimen exhibited superior performance compared to the remaining samples.

Ragunathan et al. [28] synthesized ZnO-NPs using the chemical reduction method. UV–visible spectroscopy was employed to characterize the nanoparticle, and it was determined that the plasma peak was 355.2 nm, thereby verifying the synthesis of the zinc oxide nanoparticle. The FTIR was conducted to characterize the nanoparticulate zinc oxide. The morphology of the nanoparticle was analyzed through the utilization of SEM. The nanoparticle was acquired with a round, spherical shape and a size ranging from 80 to 110 nm. Additionally, an EDX analysis was conducted, which verified that the sample consisted of 51.43 % zinc, thereby confirming the presence of ZnO-NPs. A photocatalytic investigation was conducted utilizing sunlight against methyl blue, methyl red, and Orange G. The results indicate that the highest percentage of dye decolorization after two days was observed in methyl blue, with a value of 94 %. Methyl red exhibited a lower decolorization percentage of 66 %, while Orange G demonstrated a decolorization percentage of 90 %.

Wary et al. [29] synthesized ZnO-NPs using aqueous extract from waste coconut husks. The present study confirms the synthesis of zinc oxide nanoparticles at varying pH levels in the coconut husk ash solution. The confirmation was achieved through the utilization of various analytical techniques such as powder XRD, BET analysis, SEM-EDX, UV-VIS spectroscopy, FTIR spectroscopy, and photoluminescence spectroscopy. The samples were assessed for their photocatalytic efficacy by subjecting them to solar irradiation and measuring the removal of MB and methyl orange (MO). The results indicated that the samples exhibited a removal rate of approximately 97 % and 68 % for MB and MO, respectively, within a duration of 120 min.

In our study, the results of the TGA-DTG test showed that as the temperature increases, the mass of the material decreases in the same proportion until after the formation of zinc oxide crystals at a temperature of 400° Celsius; there is no change in the mass of the material. FT-IR results indicate the formation of Zn–O bonds in all three cases of nanoparticles. In the X-ray diffraction (XRD) test, the index peaks are seen in the range of 100, 101, 002, and 110, which indicates the presence of zinc oxide, and the size of nanoparticles using Scherer's equation (1 0 1) is 32.36 nm for okra and 24.90 nm for salvia and 19.16 nm for chemical co-precipitation. The images obtained from the FE-SEM scanning electron microscope also show the hexagonal structure and the size of the nanoparticles in the A. esculentus, 40 nm, in the S. officinalis, 30 nm, and in the chemically synthesized nanoparticle is about 50 nm. The EDX results indicate the presence of O and Zn in these nanoparticles with a specific weight percentage, which shows that the total is 100 % ZnO, and no impurities were found in those nanoparticles. The photocatalytic results showed that the number of nanoparticles should be optimized for *A. esculentus* nanoparticles, 1:150 ratio, *S. officinalis* nanoparticles 1:100, and nanoparticles synthesized by chemical co-precipitation method 1:100 had the highest percentage of color degradation. So, these ratios were considered as optimal.

## 5 Conclusion

In the present study, ZnO-NPs were produced via a sol–gel procedure using *S. officinalis*, *A. esculentus*, and chemical co-precipitation methods. The synthesized nanoparticles were characterized using UV–Vis, XRD, FT-IR, DLS, zeta potential, FESEM/EDX, and TGA/DTA experiments. Their morphology and crystallite size were confirmed through the XRD and FESEM studies. The pH 10 was identified as the best pH, which had the highest percentage of color removal. Up to 360 min were tested for all three cases to optimize the dyeing time. For *A. esculentus*, the highest percentage of color removal occurred in the first 90 min, for *S. officinalis* nanoparticles between 75 and 90 min, and for chemically synthesized nanoparticles between 30 and 45 min. In conclusion, the best MB dye removal capcity belonged to co-precipitation ZnO followed by NPs synthesized using *S. officinalis* and *A. esculentus*, respectively.

## Funding

No external fund was received for conducting this study.

## Data availability

Data associated with this study can be accessed from the first author upon a reasonable request.

## CRediT authorship contribution statement

**Zakie Aalami:** Writing – original draft, Investigation, Conceptualization. **Mohammadsaleh Hoseinzadeh:** Writing – original draft, Investigation. **Parsa Hosseini Manesh:** Writing – original draft, Investigation. **Amir Hossein Aalami:** Writing – original draft, Investigation. **Zarrin Es'haghi:** Writing – review & editing, Investigation. **Majid Darroudi:** Writing – review & editing, Investigation. **Amirhossein Sahebkar:** Writing – review & editing, Investigation. **Hasan Ali Hosseini:** Writing – review & editing, Investigation.

## Declaration of competing interest

The authors declare that they have no known competing financial interests or personal relationships that could have appeared to influence the work reported in this paper.
